# Lamotrigine associated extensive hyperpigmentation: A case report and literature review

**DOI:** 10.1097/MD.0000000000039878

**Published:** 2024-10-18

**Authors:** Keling Pei, Yuqian Wu, Tao Zhang

**Affiliations:** a Pharmacy Department, Shandong Mental Health Center Affiliated to Shandong University, Jinan, China; b Pharmacy Department, Shandong Electric Power Central Hospital, Jinan, China.

**Keywords:** COVID-19, hyperpigmentation, lamotrigine, mild maculopapular exanthema

## Abstract

**Rationale:**

Lamotrigine (LTG)-associated extensive hyperpigmentation is rare and may persist for a long time or even become permanent. LTG-associated cutaneous adverse reaction (CAR) manifests initially as mild maculopapular exanthema (MPE). The first step in CAR therapy is to immediately discontinue the offending LTG for predicting evolution to mild or severe forms not always possible. Here, we present a rare case of LTG-associated extensive hyperpigmentation for delaying the withdrawal of LTG.

**Patient concerns:**

We describe the case of a female adolescent with a history of depression managed with LTG, who developed a mild MPE. Unfortunately, the patient did not discharge LTG after the occurrence of MPE until 20 days later. Then she developed a residual extensive hyperpigmentation in her trunk and extremities.

**Diagnoses:**

After a series of physical examinations and retracing past medical history, she was diagnosed with LTG-associated extensive hyperpigmentation.

**Interventions:**

The patient refused any treatment.

**Outcomes:**

Nine months later, there still existed residual hyperpigmentation in her trunk and extremities, and the range and color of hyperpigmentation have not changed significantly.

**Lessons:**

This case suggests that LTG may cause not only MPE but also extensive hyperpigmentation. When a patient displays a mild MPE following the initiation of LTG in the outpatient clinic, LTG-associated CAR should not be overlooked as a diagnosis, and early withdrawal of LTG should be considered at first.

## 1. Introduction

Lamotrigine (LTG) used in the therapy of bipolar depression^[1]^ or focal epilepsy^[2]^ has been described as a common culprit in cutaneous adverse reaction (CAR)s ranging from mild MPE to severe and life-threatening forms including Stevens–Johnson syndrome and drug reaction with eosinophilia and systemic symptoms.^[3]^ Most LTG associated CARs are T-cell mediated type IV hypersensitivity reactions^[3]^ and manifest initially as mild MPE.^[4]^ The first step in CAR treatment is to discontinue the offending LTG for predicting evolution to mild or severe conditions not always possible.^[5,6]^ A postponed diagnosis or delayed discontinuation of the culprit may cause an increased risk of severe conditions.^[7]^ We present a rare case of LTG associated extensive hyperpigmentation for delaying discontinuation the culprit in a depressed adolescent after COVID-19. Our patient pays close attention to prognosis of her skin pigmentary disorder.

## 2. Case report

**Figure 1.**
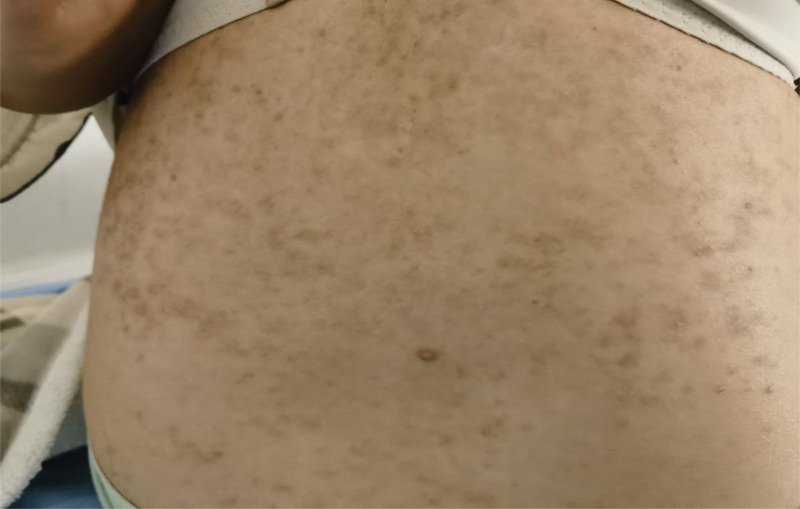
Hyperpigmentation in abdomen of the patient.

**Figure 2.**
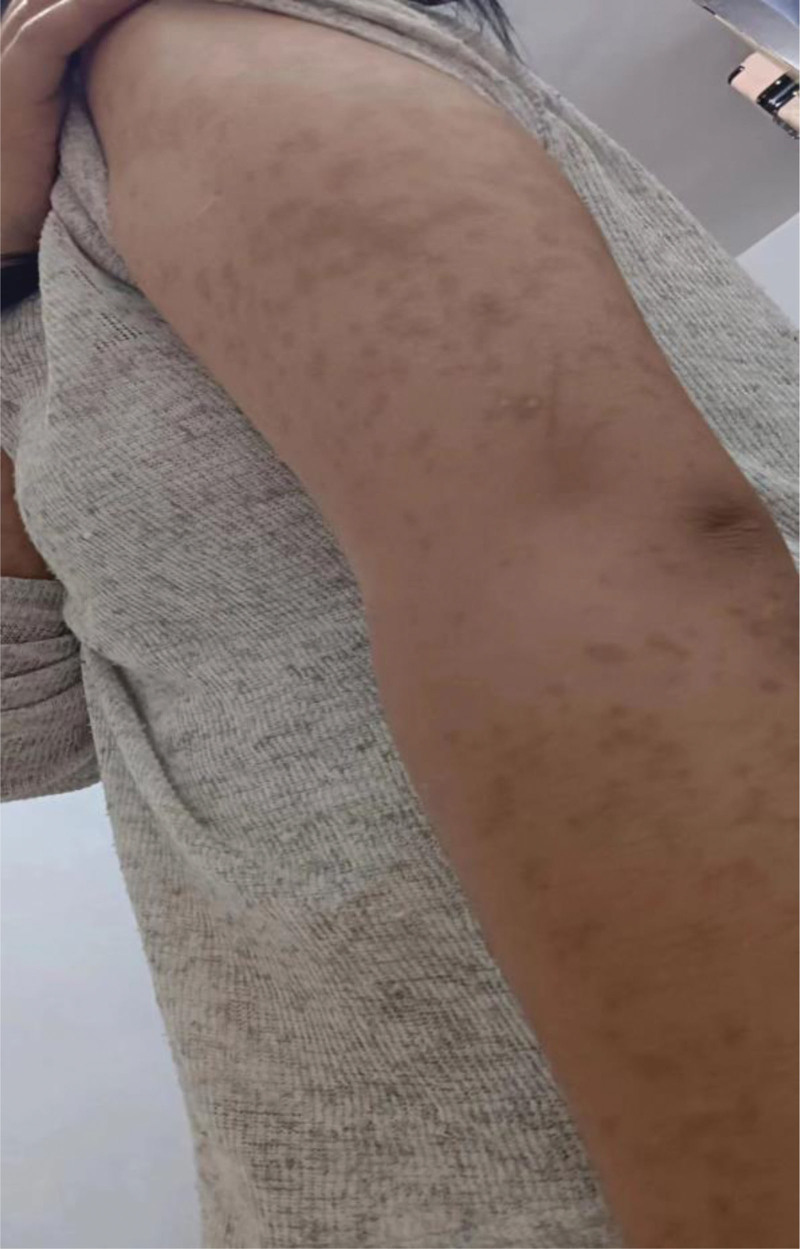
Hyperpigmentation in arm of the patient.

A female adolescent patient was admitted to our mental health ward because of loss of interest and agitation. The patient suffered from loss of interest, agitation and sleep disturbance for 2 years. Physical examination revealed extensive hyperpigmentation in her trunk and extremities. Past allergies, as evidenced by extensive hyperpigmentation in her trunk and extremities after LTG. According to detailed history, a mild MPE was more likely to occur during the first 1 week of starting LTG while infected with SARS-CoV2 at that time. Unfortunately, the patient did not receive any special treatment for SARS-CoV2. She gradually recovered from SARS-CoV2 in 15 days later, but the MPE still existed. Then she had received anti-allergy treatment for CAR in local hospital for about 4 days, but the MPE occurred repeatedly during anti-allergy treatment. Then the MPE was considered probably due to LTG, so that the LTG was discontinued and no new MPE appeared. Pruritus and MPE of the body improved greatly, but she had a residual hyperpigmentation in her trunk and extremities (Fig. 1). Then she consulted the dermatologist who recommended to avoid light without special treatment considering LTG associated extensive hyperpigmentation. Oxcarbazepine (OXC) were administered for the mild irritability during hospitalization. Her mental disorders were quickly relieved and was discharged in an emotion stable condition after 1 month of hospital stay. Nine months later after discharge, there still existed residual hyperpigmentation in her trunk and extremities, and the range and color of hyperpigmentation have not changed significantly (Fig. 2).

## 3. Discussion

CARs are a common adverse event of LTG and a major cause of LTG discontinuation.^[8]^ The incidence of benign rash caused by LTG has been estimated to 8% to 11%,^[9]^ and that of severe rash from LTG is 0.01% to 0.1%.^[10]^ The symptoms of CARs usually develop 2 to 6 weeks after taking the offending LTG but may occur at any time.^[7]^ The benign rash is generally described as mild MPE and may disappear within a few days after stopping LTG.^[11]^ In clinical practice, once a patient displays skin rash while taking LTG, the treatment for CAR is to immediately withdrawal the culprit LTG for predicting evolution to mild or severe forms not always possible. Therefore, extensive hyperpigmentation syndrome was rarely reported for mental disorder patients. Furthermore, extensive hyperpigmentation of 3 children with epilepsy caused by LTG has previously been reported to be associated with post-inflammatory hyperpigmentation and recovered slowly.^[12,13]^ Our patient developed MPE after several doses of LTG and recovered after its discontinuation leaving behind residual hyperpigmentation in her trunk and extremities. Residual hyperpigmentation lasted at least 8 months, suggesting that it is not a benign rash. To our knowledge, cases that developed extensive hyperpigmentation have not been reported previously. Our case complements this clinical information.

The pathogenesis of residual hyperpigmentation due to LTG is not well known. Histamine release and inflammatory response may be associated.^[14]^ Although the influencing factors of severe CAR of LTG are not well studied. Several studies have showed that LTG associated CARs are more frequent in adolescents than in adults, at least in the report of potentially life-threatening CAR.^[15]^ CAR develops more often in females than in males.^[15]^ What is more, females display higher CAR frequency during the reproductive years, but males suffer less frequent CAR in the same phase of life.^[15]^ The incidence of CAR is well known to be dose and titration dependent, and is closed to concomitant with valproic acid.^[16,17]^ Several other studies reported that HLA-A*24:02 alleles are possibly associated with LTG associated CAR.^[5]^

LTG associated CAR belonging to delayed-type immune reaction is mediated by T lymphocytes, and exhibits memory and cross reactivity in the clinical practice.^[18]^ Correct diagnosis of the causality of LTG-CAR is important for discontinuing the culprit, avoiding re-exposure, and finding out safe and alternative mood stabilizer.^[5]^ However another aromatic antiepileptic drug also carries a risk. LTG and OXC are similar in structure and have cross-reactions in clinic. There is no great difference in skin rash risk between patients treated with LTG and OXC.^[8]^ This risk may reduce with slow titration or desensitization regimen. A history of CAR may unjustifiably restrict future medical treatment and increase cost.^[19]^ However, most cases exposed to high-risk aromatic antiepileptic drugs did not develop cross-sensitivity.^[20]^ Luckily, our patient was given OXC to moderate her emotion without new skin rash and had a good efficacy during hospitalization.

## 4. Conclusion

Whether skin hyperpigmentation will be completely resolved after discontinuation of LTG remains undiscovered. LTG associated extensive hyperpigmentation which is rather rare and severe may be long lasting and is more distressing to patient. So far, there are no ideal treatment methods for these hyperpigmentation alterations. First, the early recognition and early withdrawal of culprit drug are important for the management of skin rash. Second, clinical timely risk assessment and targeted management will be especially required for patients with drug associated skin rash. Health care workers should be aware of this rare adverse event.

## Author contributions

**Writing – original draft:** Keling Pei.

**Writing – review & editing:** Yuqian Wu, Tao Zhang.
